# Management of percutaneous cholecystostomy drains: a survey of real-world practices across Ireland and the UK

**DOI:** 10.1186/s13017-025-00668-6

**Published:** 2026-02-21

**Authors:** Mohammed Al Azzawi, Carolyn Cullinane, Michael Devine, Stephen O’Brien, Nicola Raftery, Conor Toale, Czara Kennedy, Matthew Davey, Aine O’Neil, Noel Donlon, Jessie Elliott, William Robb, Arnold DK Hill, Jarlath Bolger

**Affiliations:** 1 Irish Surgical Research Collaborative, Royal College of Surgeons Ireland, 123 St Stephens Green, Dublin 2, Ireland; 2 Department of Surgery, Royal College of Surgeons in Ireland, 123 St Stephen’s Green, Dublin 2, Ireland; 3 Department of General Surgery, Beaumont Hospital, Beaumont Road, Dublin 9, Ireland

**Keywords:** Acute cholecystitis, Percutaneous cholecystostomy drains, Cholecystograms, Laparoscopic cholecystectomy, Open cholecystectomy

## Abstract

**Introduction:**

Acute calculous cholecystitis (ACC) is a common surgical emergency with varying severity. The Tokyo Guidelines stratified ACC into grades I-III based on severity. Patients with grade III ACC and high ASA scores can be managed with percutaneous cholecystostomy drain (PCD) insertion to control sepsis. There are currently no guidelines in the literature concerning PCD management. This questionnaire highlights the current real-life practices of PCD across Ireland and the UK.

**Methods:**

The Irish Surgical Research Collaborative sought to explore PCD practices in Ireland and the UK. This study utilised a 23-item digital questionnaire, which included questions pertaining to indications, follow-up, and scheduling of post-PCD cholecystectomy. The questionnaire was disseminated between August and October 2024 to surgical trainees and consultant surgeons from Ireland and the UK.

**Results:**

There were 94 responses from various general surgical subspecialties. Of the respondents, 61% (n = 57) were consultant surgeons, 64% (n = 60) worked in a university hospital, and 66% (n = 61) worked in a hospital without a hepatobiliary department. Forty-three Participants (46%) agreed to perform a laparoscopic cholecystectomy for ACC. However, 40% (n = 38) would insert PCD for ACC with septic shock in surgically unfit patients. Forty-six respondents (49%) chose not to perform a post-PCD cholecystogram during the index admission, and 81% (n = 76) wouldn't remove the PCD during the index admission. Regarding follow-up, forty-six participants (49%) wouldn’t perform a clamping test before PCD removal, and fifty-four would schedule an outpatient cholecystogram (57%). The majority agreed that the optimal time for a cholecystectomy is 6–12 (66%) weeks, with the laparoscopic approach (81.3%) being the most commonly chosen.

**Conclusion:**

While laparoscopic cholecystectomy remains the gold standard for managing ACC, PCDs are safe, effective, and a commonly used tool in the surgical arsenal for managing acutely unwell patients who are poor surgical candidates. Guidelines regarding management and follow-up are necessary to guide the treatment.

## Introduction

Acute calculous cholecystitis (ACC) is a gallbladder inflammation secondary to gallstones obstructing the cystic duct [1]. The majority of patients with gallstones remain asymptomatic, with a risk of 3% developing symptoms per annum [2]. Gallstone-related complications are a common surgical emergency that accounts for 20% of acute surgical presentations to the Emergency Department [1, 3]. Following an episode of acute cholecystitis, a further episode of symptomatic cholelithiasis-related complications (e.g. biliary colic, an obstructed biliary system or pancreatitis) occur in 14, 19 and 29 of patients at 6 weeks, 12 weeks and 1 year, respectively [3]. Studies have shown that laparoscopic cholecystectomy is a safe procedure [4]. The Tokyo Guidelines were first introduced in 2013 to establish the parameters for diagnosing and managing acute cholecystitis [5]. The revised 2018 version introduced a grading system to classify acute cholecystitis (grades 1–3) based on the severity of presentation and progressive organ dysfunction [6]. This classification system was established to differentiate the prognosis and treatment approach for acutely ill patients with ACC [6].

The World Society of Emergency Surgery and The Tokyo Guidelines recommend surgical management of ACC in surgically fit patients [6, 7]. The gold standard for managing ACC is laparoscopic cholecystectomy; however, it may be challenging in high-risk patients who are considered poor surgical candidates. Percutaneous cholecystostomy is regarded as a safe and suitable alternative in such instances [8]. Percutaneous cholecystostomy drains (PCD) are inserted under radiological guidance through a transhepatic or transperitoneal approach to drain the gall bladder and achieve sepsis control [9]. PCDs are deemed a safer option for managing severe acute cholecystitis (grade III) and are superior to cholecystectomy in septic patients with gangrenous and perforated cholecystitis [10–12]. Moreover, percutaneous cholecystostomy drains serve as a bridging procedure to a future cholecystectomy in acutely unwell patients and can be used as a definitive procedure in poor surgical candidates [10, 13].

Although guidelines exist regarding the role of PCDs in managing severe cholecystitis, there is a high degree of ambiguity regarding their duration, follow-up, or time of removal. Previous studies have reported a lack of consensus guidelines or established management algorithms for PCDs. This questionnaire investigates the current real-world management of PCDs in Ireland and the UK. The study aims to evaluate current practices and assess if variation exists among the surgical specialities managing PCDs.

## Methods

The Irish Surgical Research Collaborative is a trainee-led surgical research group composed of members from across Ireland. A focus group of twelve members from the ISRC was formed with variable surgical subspecialty backgrounds and at different stages of training. An iterative comprehensive literature review on percutaneous cholecystostomy drains was undertaken. The main aim of the review was to evaluate previously published literature regarding PCD and highlight the gaps in management and follow-up. Meetings were conducted to develop questions and choose the appropriate response options. The steering group reviewed and edited items for legibility; an initial draft was written. A 23-item digital questionnaire (Google Forms, Google LLC, Mountain View, CA, USA) was designed, and the study's senior authors granted the final approval. The survey was accessible online from August to October 2024, and a unique barcode was utilised for promotional purposes. Ethics application was not required for this study. Participation in the questionnaire was voluntary; thus, consent to participate was implied.

The questionnaire was divided into three main segments: *“Management of Cholecystostomy Drains During Index Admission”, “Follow-up of Cholecystostomy Drains in the Outpatient Department*”, and *“Scheduling of Completion Cholecystectomy Following Cholecystostomy Drains”.* The Department of General Surgery at the Royal College of Surgeons in Ireland emailed the questionnaire to surgical trainees and consultant general surgeons in Ireland. Reminder emails were sent periodically to improve engagement. The questionnaire was promoted by the Association of Upper Gastrointestinal Surgeons in Great Britain and Ireland, as well as the Roux Group, to UK trainees and consultants through their monthly newsletter and online platforms. The questionnaire was promoted on the ISRC official ‘X’ platform and was shared with various Irish hospitals and medical universities. It was further disseminated among consultants and trainees at local and international surgical meetings, including The Freyer Surgical Symposium and the AUGIS surgical meeting. The complete set of questions can be seen in Table 1.

**Table 1:** A survey of PCD real-world practices across Ireland and the UK: Twenty-three questions were included in the survey, which were divided into three sections

*Questions 1–11: management of cholecystostomy drains during index admission*
1. I routinely perform/attempt to perform laparoscopic cholecystectomy on acute "hot" gallbladders
2. Acute cholecystitis with septic shock is managed by inserting a cholecystostomy drain as a first-step intervention
3. Indications for a cholecystostomy drain include:
Acute cholecystitis with septic shock
Gangrenous cholecystitis
Emphysematous cholecystitis
Gallbladder perforation
Multiple medical co-morbidities
Old Age
Patients with ASA III and above
4. I only recommend cholecystostomy drains for patients who are not suitable surgical candidates
5. Access to Interventional Radiology (IR) services at my hospital:
On-site 24/7
On-site Mon-Friday during working hours
Off-site 24/7 (referral pathways available)
Off-site Monday-Friday during working hours (referral pathways available)
Not available
6. Preferred method of cholecystostomy drainage:
Under ultrasound/radiological guidance
Laparoscopic cholecystostomy
Other
7. When cholecystostomy drainage is used as a temporary measure in surgically fit patients, a cholecystectomy is performed/attempted during the index admission
8. A post procedure cholecystogram is routinely performed during the index admission as part of my practice (prior to discharge)
9. Cholecystostomy drains are removed prior to discharge if inflammatory markers and liver function tests have normalised
10. I consult a HPB surgeon for all patients who require cholecystostomy drainage
11. I routinely discharge patients with cholecystostomy drains in situ with outpatient follow up (if clinically well)
*Questions 12–18: follow-up of cholecystostomy drains in the outpatient department*
12. An outpatient cholecystogram is routinely performed prior to removal of cholecystostomy drain
13. Patients discharged with cholecystostomy drain frequently present to the A&E/readmitted with recurrence of cholecystitis or drain associated complications
14. In my practice, cholecystostomy drains are routinely removed in:
Surgical OPD
Accident & Emergency
IR following the cholecystogram
GP clinic
Surgical Assessment Unit
15. I routinely clamp the cholecystostomy tube 24–48 h prior to drain removal
16. Optimal timing of cholecystostomy drain removal (in a surgically FIT patient):
During index admission
Less than 2 weeks
Six weeks post discharge
At the time of cholecystectomy
When the drain output is minimal
17. Optimal timing of cholecystostomy drain removal (in a surgically UNFIT patient):
During index admission
Less than 2 weeks
Six weeks post discharge
At the time of cholecystectomy
When the drain output is minimal
18. I routinely contact microbiology for advice on antibiotic choice and duration for patients who require a cholecystostomy drain
*Questions 19–23: scheduling of completion cholecystectomy following cholecystostomy drains*
19. The optimal timing of a cholecystectomy in a patient with a previous cholecystostomy drain is:
During index admission
6–12 weeks after discharge
3–6 months after discharge
6–12 months after discharge
Only with recurrent cholecystitis
Drains should be kept indefinitely
20. My preferred approach to a cholecystectomy in a patient with a previous cholecystostomy drain is:
Open cholecystectomy
Laparoscopic cholecystectomy
Laparoscopic cholecystectomy with a low threshold to convert to open
21. In an acutely sick patient with septic shock secondary to acute cholecystitis, my preferred approach to management is:
Cholecystostomy drain insertion by IR
Laparoscopic / open cholecystectomy
Laparoscopy and aspiration of the gallbladder
22. Barriers to completion cholecystectomy in patients who have a cholecystostomy drain in situ:
Lack of elective theatre scheduling
Fear of procedural difficulty
Routinely refer patients to a tertiary Hepatobiliary service
Patient factors (ASA)
Patients' age
23. During an attempted laparoscopic cholecystectomy post drain insertion, there are many adhesions and difficult anatomy. My preferred approach is to:
Abandon procedure and refer to HPB
Place an intra-abdominal drain, abandon and refer to HpB
Laparoscopic subtotal cholecystectomy
Convert to open cholecystectomy
Abandon procedure and re-schedule

Ninety-four responses were sufficient to generate enough data to understand the current practice. The principal author collected and analysed the data. Responses were evaluated individually, and sub-analysis was conducted based on the responder’s speciality (Upper GI and hepatobiliary surgery versus other surgical specialities) and their country of practice (Ireland versus the United Kingdom) to assess discordance. Data were analysed using GraphPad Prism and Excel.

## Results

### Demographics of responders

Ninety-four responses were received from various general surgical subspecialties practising in Ireland and the United Kingdom. The most prevalent surgical speciality was colorectal surgery (36% %, n = 34), followed by benign upper GI surgery (18%, n = 17) and resectional upper GI surgery (13%, n = 12). Among the respondents, 60.6% (n = 57) were consultant surgeons, 6.4% (n = 6) were clinical fellows, and the remainder were surgical trainees. Thirty-seven (39%) consultant surgeons worked in the Republic of Ireland, whereas fourteen (15%) practised in the United Kingdom. There were twenty-four (26%) non-consultant doctors from Ireland and fourteen (15%) from the United Kingdom. Six hepatobiliary consultants and twenty upper GI consultants were included among the participants (28%).

**Fig. 1:**
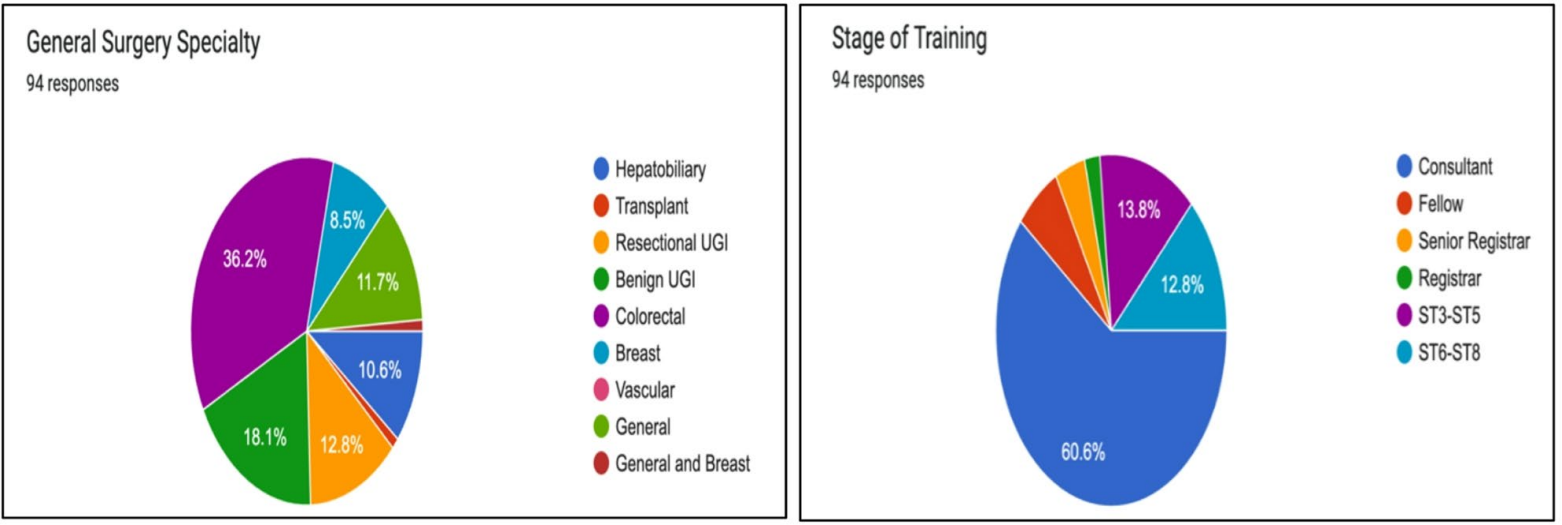
Basic demographics of the questionnaire participants. This figure illustrates the various subspecialties represented in the questionnaire and demonstrates the participants' training stages

Sixty participants (64%) worked in a Model 4 hospital, and sixty-one (66%) worked in a hospital without a hepatobiliary surgery department. Interventional radiology services were available onsite in 36% of the respondents' respective hospitals, and during the week only in 48% of the respondents' respective hospitals. Figure 1 illustrates the basic demographics of the participants.

### Management of cholecystostomy drains during index admission

Forty-three participants (46%) chose to perform or attempt laparoscopic cholecystectomy on a patient with ACC, but the lack of access to the emergency theatre remains a barrier. Similarly, most hepatobiliary and upper GI participants indicated they would perform acute cholecystectomies if their cases could be accommodated in the emergency theatre. There was a lack of consensus regarding using cholecystostomy drains as a first step in managing patients with acute cholecystitis and septic shock. Thirty-eight (40.4%) of the participants agreed, thirty-four (36.2%) disagreed, and the remainder were neutral. The same trend was evident among the hepatobiliary and upper GI speciality respondents. However, 61.3% of participants would insert a cholecystostomy drain for a patient with a septic shock secondary to acute cholecystitis.

**Fig. 2:**
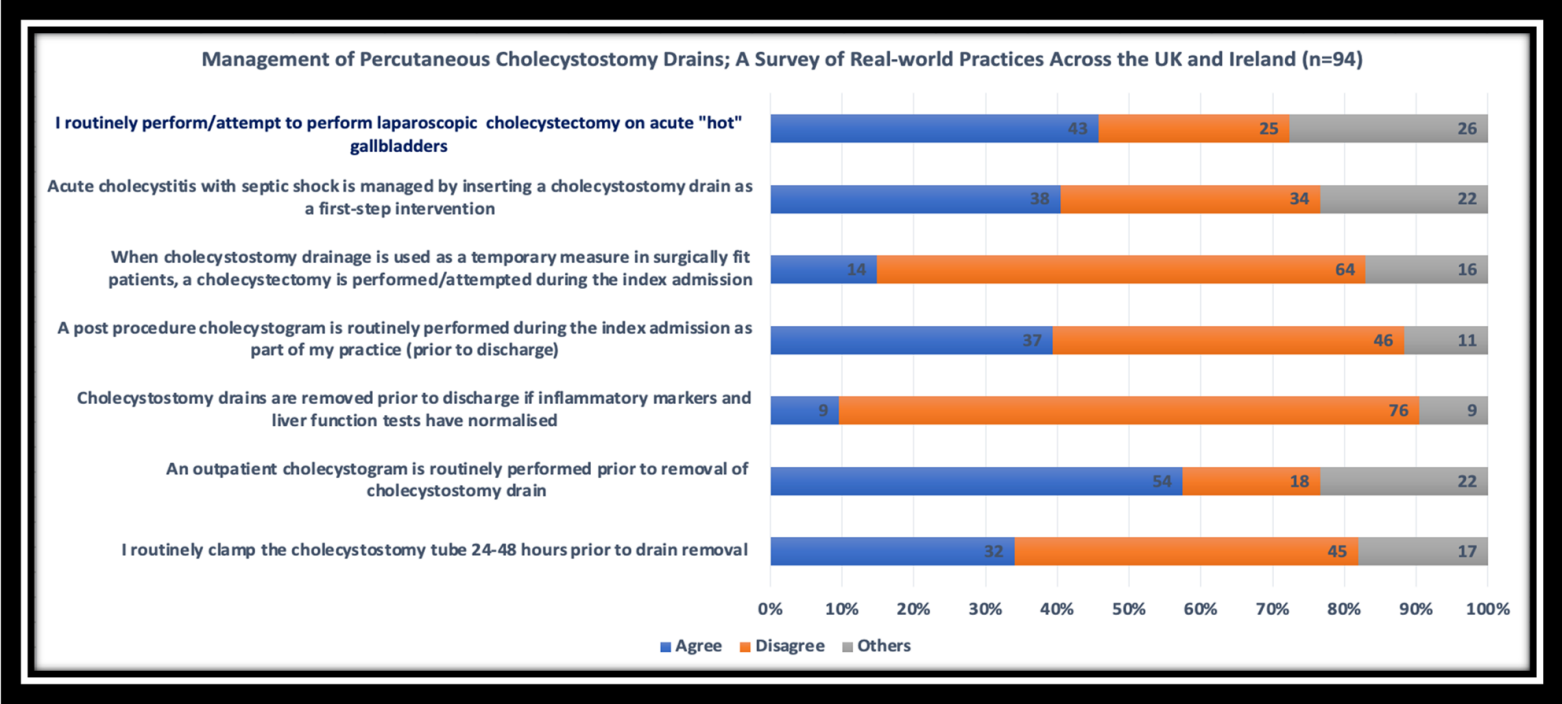
Management of percutaneous cholecystostomy drains questionnaire. The figure illustrates a sample of the questionnaire's questions and responses. A total of 94 responses were recorded during the process

The most common indication for PCD insertion was in comorbid patient (21%), followed by acute cholecystitis with septic shock (19%) and gallbladder perforation (13%). Seventy-six participants (81%) chose not to remove the cholecystostomy drain during the index admission, even if the liver enzymes and inflammatory markers had normalised. Forty-six participants (49%) would not perform a post-procedure cholecystogram, and only 12% (n = 12) would consult the hepatobiliary service to manage cholecystostomy drains. A sample of questions related to indications, follow-up, and post-PCD cholecystectomy, along with their respective answers, can be found in Fig. 2.

### Follow-up of cholecystostomy drains in the outpatient department

Most participants (94%, n = 88) were willing to follow up with patients with PCDs in the outpatient department. Forty-six participants (49%) felt that most patients do not present with drain-related complications to the A&E following discharge. There was a consensus (57%) on performing an outpatient cholecystogram before removing cholecystostomy drains. This view was also supported among participants with hepatobiliary and upper GI specialities (25 out of 26). In contrast, 48% chose not to perform a clamping test before removing cholecystostomy drains. Participants with HpB and UGI backgrounds expressed differing opinions on the necessity of a clamping test, with 18 opting to perform the test compared to 16 who considered it unnecessary. The most frequently selected option for the optimal timing of drain removal was six weeks post-discharge (42%), followed by “at the time of cholecystectomy” (33%) for a surgically fit patient. For surgically unfit patients, the most commonly selected options for drain removal were six weeks post-discharge (43%) and indefinite retention (20%).

### Scheduling of completion cholecystectomy following cholecystostomy drains

**Fig. 3:**
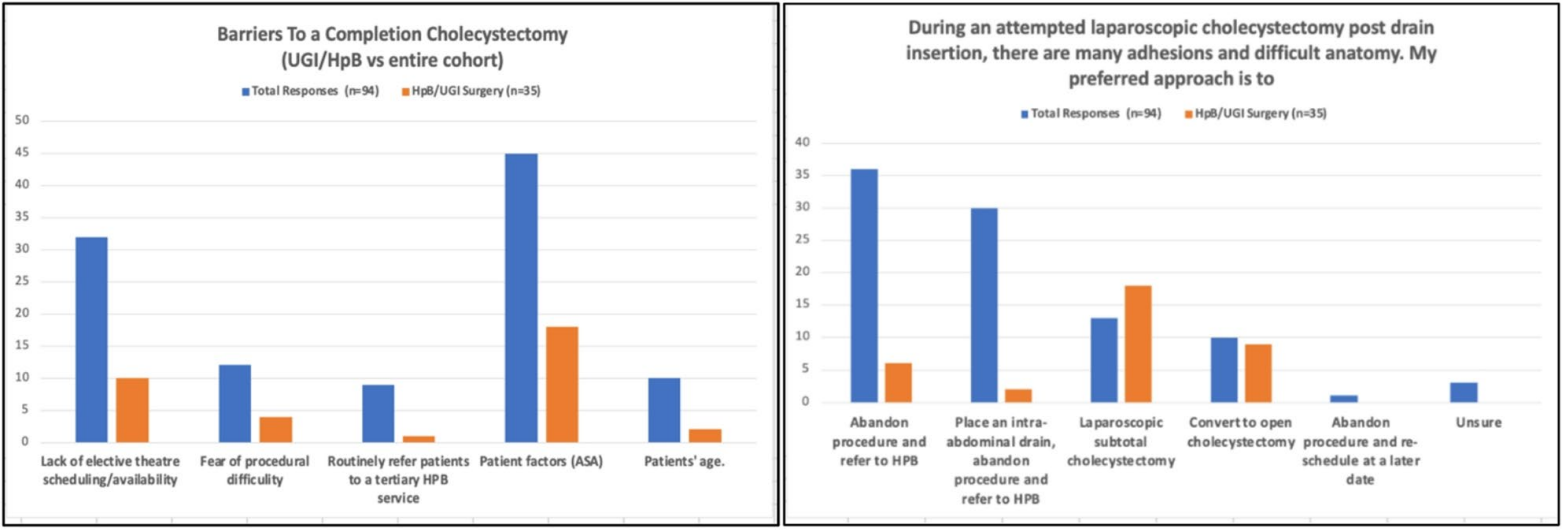
Discrepancy between UGI/HpB and the entire cohort in performing a completion cholecystectomy: The figure illustrates the differences between the HpB/UGI responses (n = 35) and the whole cohort responses (n = 94)

Around two-thirds of the participants (68%) indicated that they would not perform a laparoscopic cholecystectomy after PCD insertion in a surgically fit patient during the index admission. This was similar among the Irish and British cohorts and participants with a hepatobiliary and upper GI background. Sixty-three participants (67%) would attempt a cholecystectomy within six to twelve weeks of PCD insertion, with laparoscopy being their preferred surgical initial approach (81%). These were also the most commonly selected options in the hepatobiliary and upper GI surgery groups. The primary barriers to completing a cholecystectomy following PCD insertion included patient comorbidities/ASA score (42%), lack of elective theatre availability (30%), and fear of procedural difficulty (11%). One-third of participants (34%, n = 32) chose to abandon the procedure if they encountered a difficult cholecystectomy post-PCD insertion. In comparison, 32% (n = 30) opted for a subtotal cholecystectomy, and 12% (n = 11) would convert to an open cholecystectomy. Figure 3 illustrates the difference in answers between the general surgery consultants and UGI/HpB consultants when asked about barriers to a completion cholecystectomy.

## Discussion

Percutaneous cholecystostomy drainage is a safe and effective procedure for managing acutely unwell patients with severe calculous cholecystitis [6, 10, 14]. Our study aimed to evaluate the current real-world practices of PCDs and their follow-up. Our participants concurred on the necessity of performing a laparoscopic cholecystectomy for ACC; however, the lack of access to emergency theatres remains an obstacle. Therefore, PCDs are accepted as alternatives for severe ACC with concomitant septic shock. Regarding PCD follow-up, an outpatient cholecystogram was routinely performed as part of the follow-up in the Irish and the UK cohorts, and the ideal time for drain removal was four to six weeks. This was to ensure tract maturation and avoid drain-related complications. The optimal time for a completion cholecystectomy following PCD insertion was six to twelve weeks. The main challenge for performing completion cholecystectomy was the lack of access to theatres among the Irish and British cohorts, leading both to opt for PCDs as a definitive procedure in surgically unfit patients.

While laparoscopic cholecystectomy remains the gold standard for treating acute cholecystitis, patient comorbidities, fear of operative difficulties in the acute setting, and lack of access to emergency theatres pose significant obstacles [15, 16]. The Tokyo Guidelines 2018 recommend the use of PCDs for managing unfit patients with severe grade III ACC [6, 10]. However, no consensus guidelines exist to date for managing and following up on cholecystostomy drains. The role of PCDs revolves around managing acutely unwell patients with septic shock, localised perforation, and comorbid patients, similar to our study findings [17, 18]. The lack of access to emergency theatre, the severity of clinical presentation, and the fear of technical difficulties intraoperatively are some of the main reasons PCDs are preferred over laparoscopic cholecystectomy in the acute setting [17–19].

In terms of follow-up investigations, previous studies have shown that clamping tests are associated with a lower rate of ACC recurrence, whereas performing a cholecystogram before PCD removal is not [19, 20]. However, cholecystograms are routinely performed to follow up PCD and ensure tract maturation prior to drain removal [20]. They are especially indicated if liver enzymes were deranged or if there was evidence of choledocholithiasis at the time of PCD insertion [20]. In our study, participants with Hepatobiliary and upper GI surgery backgrounds advocated for clamping tests before PCD removal; this was not a routine procedure by other specialities.

In terms of drain removal, previous studies have shown that the ideal time for cholecystostomy drain removal was within four to six weeks of insertion, similar to our study findings [10, 21]. However, the timing of drain removal depends on the insertion method [22]. Transhepatic drains can be removed safely earlier than transperitoneal drains and have a lower complication rate than transperitoneal drains [22]. A completion cholecystectomy following PCD insertion remains a controversial topic in terms of ideal timing and fear of complications. However, the literature has shown that it is a safe procedure, characterised by a low complication rate and a minimal incidence of conversion to open surgery [23, 24]. The optimal timing for a cholecystectomy is six to twelve weeks after PCD insertion, which aligns with our study findings. Our study highlighted the main barriers to completion of cholecystostomy, including a lack of access to elective operating theatres, fear of procedural difficulty, and a poor ASA score. Participants with non-HpB and UGI backgrounds opted for abandoning the procedure and placing a drain or referring to an HpB surgeon for cholecystectomy. This primarily revolves around the medical background and clinical presentation of the patients receiving a PCD. They tend to be comorbid, elderly, with a poor functional baseline and a severe onset of ACC [2, 3]. These obstacles were similar amongst the Irish and British cohorts and were also similar in those with HpB/UGI background compared to other specialities. While cholecystostomy drains are classically used for managing poor surgical candidates with septic shock secondary to ACC, there are new emerging techniques, such as EUS-guided drainage (LAMS) using the Axios stent, to manage poor surgical candidates who won’t end up with a completion cholecystectomy [25].

Our study aimed to evaluate the current real-world practice of managing cholecystostomy drains in Ireland and the UK while highlighting the lack of guidelines in the existing literature. The survey findings are limited by the participants’ experience and current practice and may not represent the standard of care throughout Ireland and the UK. The study highlighted the heterogeneity in practice rather than providing guidelines for managing PCDs. Therefore, future studies should aim to develop guidelines for the management and follow-up of cholecystostomy drains.

## Conclusion

Cholecystostomy drains are a commonly used tool in the surgical arsenal for managing acutely unwell patients who are poor surgical candidates. There is a high heterogeneity in practice regarding the insertion and follow-up of PCDs. Guidelines regarding their indications, management, and follow-up are necessary to guide the treatment.

## Data Availability

I'm happy to provide the data included in the study.
